# Invasive Fungal Rhinosinusitis Due to Co-infection with Mucormycosis and *Exserohilum rostratum* in a Patient with Acute Lymphoblastic Leukemia

**DOI:** 10.1007/s44228-022-00009-3

**Published:** 2022-06-13

**Authors:** Vera Radici, Eolia Brissot, Suzanne Chartier, Juliette Guitard, Bettina Fabiani, Mara Memoli, Anne Banet, Laurence Heuberger, Simona Lapusan, Sarah Atallah, Ollivier Legrand, Alexis Genthon

**Affiliations:** 1 Service d’Hématologie Clinique et Thérapie Cellulaire, Hôpital Saint-Antoine, Sorbonne Université, AP-HP, Paris, France; 2 Clinical Hematology and Bone Marrow Transplant Centre, S.Maria della Misericordia University Hospital, University of Udine, Udine, Italy; 3 Service d’Anatomie Pathologique, Sorbonne Université, AP-HP, Hôpital Saint-Antoine, 75012 Paris, France; 4 Sorbonne Université, Inserm, Centre de Recherche Saint-Antoine, CRSA, AP-HP, Hôpital Saint-Antoine, Service de Parasitologie-Mycologie, 75012 Paris, France; 5 Department of Medicine and Surgery, Hematology and Hematopoietic Stem Cell Transplant Center, University of Naples Federico II, Naples, Italy; 6 Service d’Hématologie, Centre Hospitalier de la Polynésie Française, Pirae, Tahiti France; 7 Department of ENT-Head and Neck Surgery, Tenon Hospital, APHP, Sorbonne University, 4 Rue de La Chine, 75020 Paris, France

**Keywords:** *Exserohilum rostratum*, Setosphaeria, Mucormycosis, Invasive fungal infections, Acute lymphoblastic leukemia

## Abstract

Invasive fungal infections remain an important cause of complication and morbidity in the management of acute leukemias. Here we report the case of a 27-year-old patient from French Polynesia who was diagnosed with Philadelphia chromosome-negative B-cell acute lymphoblastic leukemia. After induction chemotherapy, she developed rhinosinusitis with extensive bone lysis. The context and clinical presentation quickly made us suspect an invasive mucormycosis infection. However, a multidisciplinary investigation including mass spectrometry techniques also revealed the presence of *Exserohilum rostratum*, a pathogen member of the genus *Exserohilum* that is ubiquitous in tropical and subtropical regions but rarely implicated in invasive sinusitis. Antifungal treatment combined with an early surgical approach resulted in a favorable clinical response.

## Introduction

Invasive fungal infections (IFIs), including those caused by various Mucorales species [1], such as *Mucor, Rhizopus* and *Lichtheimia,* and other newly emerging fungal species are important causes of morbidity and mortality in immunocompromised patients. Mucormycosis is a serious but rare, fungal infection, with high morbidity and mortality. The most common clinical presentations are rhino-orbito-cerebral, pulmonary, cutaneous, and disseminated.

The genus *Exserohilum* (teleomorph *Setosphaeria*), comprising approximately 35 species, is a common saprophytic fungus of plants in warm and humid climates [2]. They occasionally cause infections in plants and animals, and can rarely be pathogenic for humans, mostly in tropical and subtropical regions. The most described infections are sinusitis, keratitis, skin, and subcutaneous infections, although brain abscesses, osteomyelitis, endocarditis, and disseminated infections have also been reported, either by direct inoculation or by dissemination in immunocompromised patients [3].

## Case Report

A 27-year-old French Polynesian female patient was initially treated in February 2019 for what was thought to be diffuse large-cell B lymphoma. The patient was in complete remission after eight cycles of R-CHOP (comprising rituximab, cyclophosphamide, doxorubicin, vincristine, and prednisone). Nine months later, a relapse was suspected due to the development of a renal mass. The new histological examination was in favor of an extramedullary localization of acute lymphoblastic leukemia (ALL). The myelogram revealed a blast infiltration, and the diagnosis of B-ALL was made. Induction chemotherapy was started on October 2020 following the GRAAL-2014 protocol (including vincristine, cyclophosphamide, L-asparaginase and methylprednisolone). Two weeks later, a palatine median filamentous ulceration appeared, macroscopically whitish, raised, asymmetrical and indolent, which was initially managed with amphotericin B mouth rinses. Following the next course of chemotherapy (high-dose methotrexate), the lesion did not regress.

**Fig. 1:**
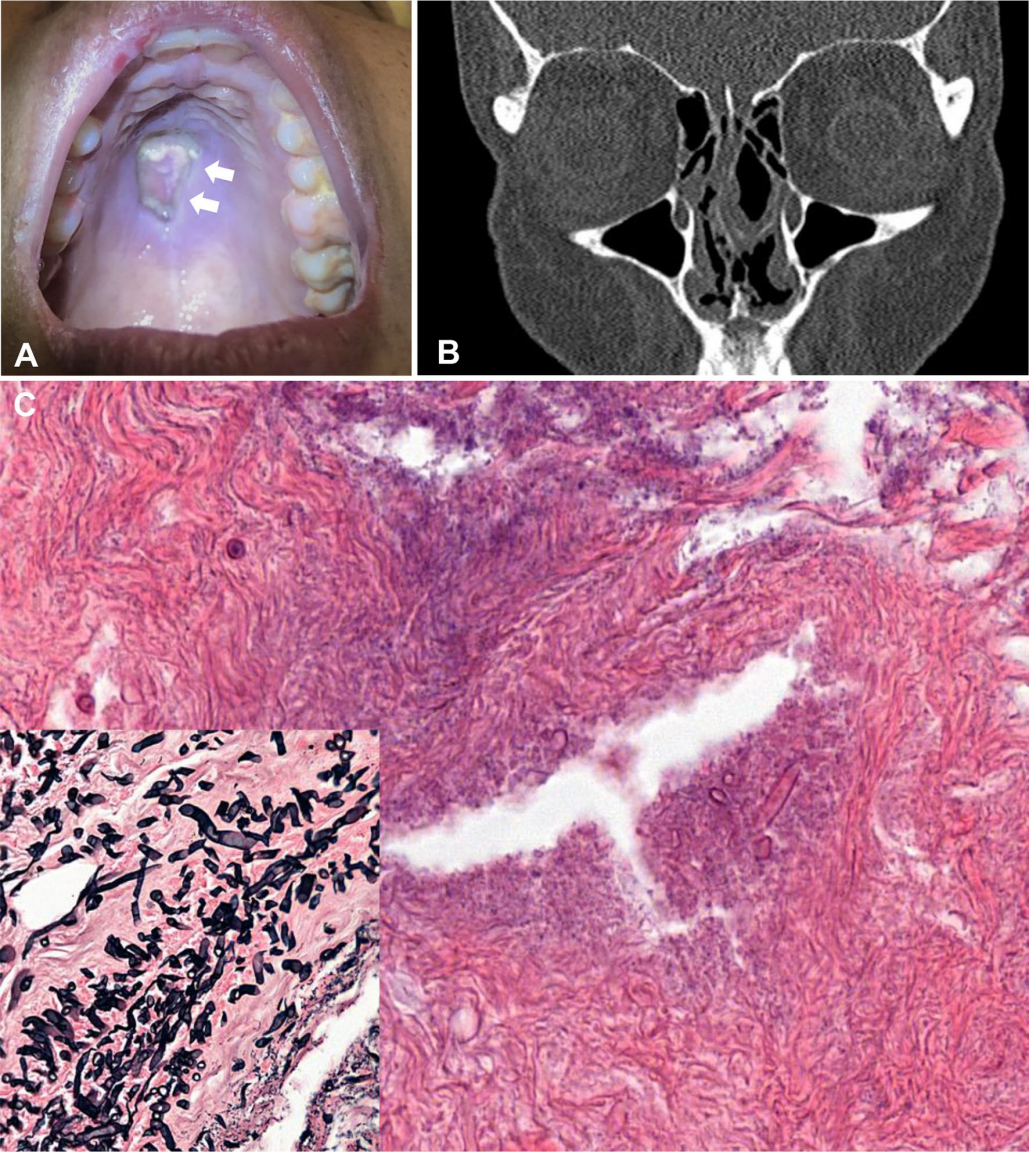
Initial palatine lesion (**A**, withe arrow) and CT scan before surgery showing palatine and septum lysis (**B**). Massive necrosis of the palate biopsy (**C**), hyphae identified by routine hematoxylin-eosin stain broad and ribbon-like hyphea in blood vessel stained with Grocott (**C**, insert)

Biopsy of the palate ulceration showed mucosal necrosis with fungal filaments within the adipose tissue and vessels (Fig. 1A), Grocott staining- highlighted large minimally-septated hyphae, penetrating blood vessels, suggestive of mucormycosis (Fig. 1C). A computed tomography (CT) scan of the maxillary sinus documented an erosive aspect of the lower part of the nasal septum and the anterior part of the bony palate (Fig. 1B). Nasal endoscopy showed an extensive necrosis of the maxillary floor, the septum wall and extension to the skull base. Intravenous liposomal amphotericin B was initiated at 5 mg/kg/day, associated with two surgical debridements of the necrotic areas. The first consisted of a transnasal necrosectomy endoscopic surgery. An inferior partial maxillectomy, septum resection to the olfactory cleft and an anterior ethmoidectomy was performed, thereafter. Several biopsies were realized and analyzed. Biopsies were performed at the mucosal extremities of the debridement.

Direct examination of the nasal necrosis showed numerous mycelial hyphae of unknown origin. Molecular detection (in-house PCR) of Mucorales was positive in three different biopsy specimens (crossing point between 35 and 38). Serum Mucorales detection remained negative during follow-up. Molecular detection of *Aspergillus fumigatus*, galactomannan antigen, *Candida* antigen and Beta-D-Glucan in serum were all negative. Surprisingly, biopsy cultures returned positive with a mold, identified as *Exserohilum rostratum* using mass spectrometry (MSI software, and 18S and 28S rDNA sequencing). EUCAST MIC determination was carried out and this isolate was susceptible to amphotericin B, but with elevated minimum inhibitory concentration to voriconazole and isavuconazole (Table 1). Specificity of our Mucorales PCR was confirmed, as a negative amplification was obtained with DNA extracted from the *E. rostratum* isolate.

**Table 1:** Determination of antifungal susceptibility of *E. rostratum* isolates using European Committee on Antimicrobial Susceptibility Testing (EUCAST) method

	AMB		VOR		ITR		ISA		POS	
Time	24 h	48 h	24 h	48 h	24 h	48 h	24 h	48 h	24 h	48 h
MIC (μg/mL)	0.03	0.25	0.03	2	0.016	0.125	1	8	0.03	0.25

*AMB* amphotericin B, *VOR* voriconazole, *ITR* itraconazole, *ISA* isavuconazole, *POS* posaconazole,* MIC* Minimal inhibitory concentration

Post-surgery magnetic resonance imaging (MRI) of the sinuses showed the presence of a necrotic lesion of the nasal septum compatible with a mycotic origin, with massive osteolysis of the bony palate, but no extension to the anterior stage of the base of the skull or any element of pachymeningitis. A total body scanner documented the presence of spontaneous hyperdense filling centered on the nasal septum, with lysis of the latter and of the bony palate extending to the anterior part of the maxilla on the median line opposite the dental roots of incisors 11 and 21. There was no basis for suspecting an extra-cranial fungal localization. To complete the debridement of the necrotic areas, sphenoidectomy and bilateral ethmoidectomy were performed, and a mobile palatine prosthesis was constructed to allow the patient to eat. The macroscopic result was satisfactory with no extension of the necrotic areas.

MRI and CT control scans were performed one month after surgery and did not show any evolution of the co-infections. At the otorhinolaryngology evaluation, perfect mucosal healing was confirmed without clinical indication of disease progression. After 7 weeks of treatment with liposomal amphotericin B, fungal treatment was switched to isavuconazole.

The patient is currently in good clinical condition,with no signs of progression of the known infection. She was able to continue her therapy for leukemia.

## Discussion

IFIs most frequently affect immunocompromised patients, especially those with hematological disease [4]. Despite advances in diagnosis and management, IFIs remain a major cause of high mortality and morbidity. Timely diagnosis and early initiation of antifungal therapy are considered essential for survival of these patients. Our patient developed a palatine lesion with no further symptoms, and the combination of radiological images, lesion characteristics, and the presence of fungal filaments led to early initiation of antifungal treatment. At the time of clinical presentation, several fungal infections were under consideration, such as aspergillosis or mucormycosis. Mucormycosis was then confirmed by molecular diagnosis on the first biopsy performed on the palatine mucosa. Unexpectedly, the culture from biopsy samples identified *E. rostratum*. In this complex scenario, a proven mould infection was diagnosed according to the criteria set by the European Organization for Research and Treatment of Cancer (EORTC) [1], and attributed to, probably, mucormycosis and *E. rostratum*.

Upon suspicion of mucormycosis, appropriate imaging to document the extent of disease, followed by surgical resection are strongly recommended. First-line treatment with high-dose liposomal amphotericin B is usually proposed; indeed, our patient was promptly started on the antifungal therapy and underwent two surgical debridements within 48 h. An 8-week antifungal treatment resulted in complete resolution of the infection at the clinical and imaging level. It was then decided to start treatment with isavuconazole, after discontinuing liposomal amphotericin B, based on experimental animal model which demonstrated antifungal efficacy against *E. rostratum* [5] and known anti-Mucorales activity [6]. Although experts agree on primary prophylaxis of these infections, there are no guidelines on secondary prophylaxis.

The diagnosis of a rhinosinusoidal infection by *E. rostratum* was confirmed in this French Polynesian patient. The members of the heat-tolerant genus *Exserohilum* are ubiquitous in tropical and subtropical regions. However, less than ten cases of invasive non-allergic sinusitis due to *Exserohilum* have been described in the literature in haematological patients, who were typically pediatric and from warm areas [7–11]. In 2012, this fungus was the cause of an outbreak of meningitis associated with contaminated methylprednisolone injections [12]. In this context, susceptibility to liposomal amphotericin B was demonstrated.

It is essential to perform a lesion biopsy and adequate imaging in hematological patients in whom we strongly suspect a fungal infection, in order to start treatment promptly. An aggressive multidisciplinary approach must be taken, comprising experts in oral surgery, infectious disease, microbiology, radiology and pathology. Interestingly, the fast and favorable outcome in our patient allowed the continuation of the ALL treatment.

In conclusion, this is the first case report of invasive fungal rhinosinusitis co-infection due to Mucorales and *E. rostratum* in a French Polynesian patient with high-risk ALL.
